# How have researchers defined and used the concept of ‘continuity of care’ for chronic conditions in the context of resource-constrained settings? A scoping review of existing literature and a proposed conceptual framework

**DOI:** 10.1186/s12961-019-0426-1

**Published:** 2019-03-07

**Authors:** Lana Meiqari, Tammam Al-Oudat, Dirk Essink, Fedde Scheele, Pamela Wright

**Affiliations:** 1 Athena Institute for Research on Innovation and Communication in Health and Life Sciences, Faculty of Sciences, Vrije Universiteit Amsterdam, De Boelelaan 1085, 1081 HV Amsterdam, The Netherlands; 2 Department of Public Health, Institute of Tropical Medicine, Antwerp, Belgium; 3 Médecins Sans Frontières, Operational Centre Geneva (MSF-OCG), Geneva, Switzerland; 4 Guelph International Health Consulting, Amsterdam, The Netherlands

**Keywords:** Continuity of care, long-term care, chronic care, resource-constrained setting, primary healthcare, health policy and systems

## Abstract

**Background:**

Within the context of the growing burden of non-communicable diseases (NCDs) globally, there is limited evidence on how researchers have explored the response to chronic health needs in the context of health policy and systems in low- and middle-income countries. Continuity of care (CoC) is one concept that represents several elements of a long-term model of care. This scoping review aims to map and describe the state of knowledge regarding how researchers in resource-constrained settings have defined and used the concept of CoC for chronic conditions in primary healthcare.

**Methods:**

This scoping review adopted the modified framework for interpretive scoping literature reviews. A systematic literature search in PubMed was performed, followed by a study selection process and data extraction, analysis and synthesis. Extracted data regarding the context of using CoC and the definition of CoC were analysed inductively to identify similar patterns; based on this, articles were divided into groups. MaxQDA was then used to re-code each article with themes according to the CoC definition to perform a cross-case synthesis under each identified group.

**Results:**

A total of 55 peer-reviewed articles, comprising reviews or commentaries and qualitative or quantitative studies, were included. The number of articles has increased over the years. Five groups were identified as those (1) reflecting a change across stages or systems of care, (2) mentioning continuity or lack of continuity without a detailed definition, (3) researching CoC in HIV/AIDS programmes and its scaling up to support management of NCDs, (4) researching CoC in NCD management, and (5) measuring CoC with validated questionnaires.

**Conclusion:**

Research or policy documents need to provide an explicit definition of CoC when this terminology is used. A framework for CoC is suggested, acknowledging three components for CoC (i.e. longitudinal care, the nature of the patient–provider relationship and coordinated care) while considering relevant contextual factors, particularly access and quality.

**Electronic supplementary material:**

The online version of this article (10.1186/s12961-019-0426-1) contains supplementary material, which is available to authorized users.

## Introduction

Improved health systems have been essential in efforts to achieve the Millennium Development Goals of reducing child and maternal mortality and combating HIV/AIDS and other communicable diseases; these health improvements have resulted in higher life expectancy and ageing populations worldwide [1]. Nevertheless, in many low- and middle-income countries (LMICs) the burden of communicable, maternal, neonatal and nutritional disorders has remained a priority, while a growing burden of non-communicable diseases (NCDs) and injuries has concurrently emerged as a new challenge [2]. In the era of Sustainable Development Goals, strategies and policies proposed to strengthen health systems to cope with this double burden include achieving universal health coverage, prioritising primary and community care services, and strengthening referral systems, along with efforts towards patient empowerment. However, implementing such policies in LMICs demands a radical paradigm shift in how health services are managed, delivered and funded [3]. For example, health services previously organised to provide care for acute and episodic conditions now need to provide long-term care across disciplines to manage chronic diseases effectively, efficiently and cost-effectively [4, 5].

The most common conceptualisation of designing and providing care for chronic conditions is the Chronic Care Model (CCM) developed by Wagner in the 1990s [6]; the CCM emphasised the productive interaction of the “*informed and activated*” patient with the “*prepared and proactive*” healthcare team, which will lead to improved health outcomes. The CCM argued that a productive patient–provider interaction needs a comprehensive system change in the organisation of primary healthcare in terms of resources and policies, self-management support, decision support, delivery system design and clinical information systems [6]. This model was adopted and expanded to the global level through WHO’s Innovative Care for Chronic Conditions (ICCC) framework, which suggested building blocks at three levels of the health system (i.e. community, healthcare organisation and positive policy environment), “*that can be used to create or re-design a healthcare system to more effectively manage long-term health problems*” [7]. At the healthcare organisation level, the building blocks were to promote continuity and coordination, encourage quality through leadership and incentives, organise and equip healthcare teams, use information systems, and support self-management and prevention [7]. Over the years, several entangled concepts and frameworks have been used to define essential elements of a long-term model of care for research and policy at both global and national levels, including continuity or coordination or integration of care, patient- or people-centred care, case management, continuum of care, and holistic care [3, 6, 7]. A literature review identified three core values and themes shared by these concepts, namely (1) personal relationship between patient and provider, (2) communication of relevant information between providers, and (3) cooperation between providers within and between healthcare settings [8].

However, all of these concepts and frameworks, including CCM, were defined and designed based on research in high-income countries. There is scarce evidence on how the response to chronic health needs has been translated into the context of LMICs, which face additional challenges such as weak health systems and constrained resources. Furthermore, researchers and policy-makers are increasingly recognising that there is no ‘one-size-fits-all’ or ‘one model’ for health interventions [3]; therefore, differences in contexts, populations, and even times must be considered when applying health concepts and interventions in research and policy [9, 10]. There have been several attempts to adapt models of chronic care to the context of LMICs. For example, a recent article synthesised evidence from primary qualitative research and found that all themes of CCM were included in the analysed studies, in addition to four further themes, namely quality of communication between health professionals and patients, availability of essential medicines, diagnostics and trained personnel at decentralised levels of healthcare, and mechanisms for coordination between healthcare providers [11]. Another article used South Africa as a case study to analyse the application of the ICCC Framework in light of multimorbidity due to comorbid NCDs and infectious chronic diseases, and highlighted the importance of patient perspectives, experiences and capacity that contribute to better treatment adherence, healthcare utilisation and health outcomes.

One concept which is driven by the patient perspective and their sense of their relationship with the healthcare provider and the health system is ‘continuity of care’ (CoC) [12]. Different definitions and perspectives for CoC have been identified by research conducted in high-income countries (Additional file 1). Globally, the ICCC Framework introduced the element of continuity as care “*planned and thoughtful over the course of the condition*” [7], which emphasised the role of organisations in providing proactive care, including scheduled follow-up visits. Later, WHO adopted a definition of CoC as “*the degree to which a series of discrete health care events is experienced by people as coherent and interconnected over time, and consistent with their health needs and preferences*” [3]. This definition focuses on CoC as a process experienced by patients accessing care that fulfils their health needs and leads to better outcomes, such as higher patient satisfaction and quality of life, improved delivery of preventive services, fewer visits to emergency departments, and lower hospitalisation rates [13–16]. From the perspective of complex systems, CoC became a benchmark for quality of care [17], and subsequent research often focused on CoC as an outcome for ongoing complex interactions within the health system, aimed at finding evidence on how better to achieve CoC [10]. In general, CoC definitions share three common themes or elements, namely (1) care delivered over time with as few doctors as possible (i.e. longitudinal care), (2) a caring relationship between patients and health professionals (i.e. patient–professional relationship), and (3) cooperation and communication of relevant information between providers within and between care settings (i.e. coordinated care).

To further synthesise available evidence on chronic care in resource-constrained settings as experienced by patients and providers, we decided to focus on the concept of CoC. We aim to map and describe the state of knowledge regarding how researchers in resource-constrained settings defined and used definitions of the concept of CoC for chronic conditions in primary healthcare. This article uses a scoping review methodology as it aims to clarify a complex concept with an exploratory review of the literature [18, 19].

## Methods

This scoping review adopted Daudt et al.’s modifications [20] to Arksey and O’Malley’s framework for interpretive scoping literature reviews [21], using the five steps of identifying the research question, identifying relevant studies, study selection, charting the data, and collating, summarising and reporting the results.

### Identifying the research question

Given the aim of this review, the following research questions were formulated:

1. What type of research used the concept of CoC in primary healthcare in resource-constrained settings?
2. What definitions and/or frameworks for CoC were used?
3. What measurements for CoC were used?

### Identifying relevant studies

A literature search was performed in PubMed using relevant keywords and index terms. Search terms were developed following the elements of population, concept and context. The population under study is composed of patients who have chronic conditions and need long-term care, including those with chronic NCDs or communicable diseases. The concept is CoC at the primary healthcare level. The context is resource-constrained settings (i.e. LMICs). Detailed tables on the search terms used are presented in Additional file 2. The search covered existing publications, reporting quantitative and qualitative results, in English, with no time restrictions.

### Study selection

One researcher (LM) screened titles and abstracts using the Rayyan QCRI application to choose relevant records [22]; full texts were obtained and read to assess each study for inclusion, based on the criteria that papers described the need or use of CoC in primary healthcare in resource-constrained settings, without limitations to the type of study design, accepting primary research studies, systematic reviews, meta-analyses and editorials.

This review focuses on the use of definitions and/or frameworks for data collection or in writing the article; it does not attempt to summarise evidence for any outcome or measurement. Thus, no quality assessment was performed during the process of selection.

### Charting the data

Data extraction, analysis and synthesis involved an iterative process of reading and re-reading the selected studies. Essential information was extracted into a standardised spreadsheet, including author, publication year, research aim/objective, country, disease or population under investigation, research design, context for using CoC, CoC definition, framework, measurement (when relevant), and results.

### Collating, summarising and reporting the results

Firstly, a thematic analysis was used in which extracted data regarding the context of using CoC and CoC definition were analysed inductively to identify similar patterns. Based on the identified themes, articles were divided into groups. Secondly, MaxQDA was used to re-code each article with themes focused on CoC definition and its three items of longitudinal care, patient–professional relationships, and coordinated care. Consequently, we performed a cross-case synthesis for the articles within each group to compose the findings.

## Results

### Study selection

The database search produced 2116 records, of which 1982 were excluded by title/abstract screening. Full texts of the remaining 134 records were examined and those that did not use or define CoC were discarded. Finally, 55 peer-reviewed articles were selected for review.

### Study characteristics

An overview of each article is available in Table 1. While all identified articles were published in the new millennium, the number of articles increased from 1 article in 2002–2005 to 2–5 articles per year in the following years, jumping to 19 articles in 2015 (34.6%).

**Table 1:** Study characteristics of included peer-reviewed articles (*n* = 55), divided by the five groups identified based on their use of the ‘continuity of care’ concept

First author, year	Study type	Condition	Location^a^	Main focus of the article
Group 1: Reflecting a change in stages or systems of care				
Robles, 2004 [28]	Review	Chronic diseases	Americas	A public health framework for chronic disease prevention and control
Mayige,2011 [25]	Review	NCDs	Tanzania	NCD services
Pakdeeprom, 2012 [30]	Cross-sectional survey	Chronic diseases	Thailand	Transition from paediatric to adult care system for patients with chronic illnesses
Ichiho, 2013 [23]	Review and assessment	NCDs	Federated States of Micronesia	Systems perspective on NCDs, including diabetes
Armstrong, 2014 [35]	Case study	Tuberculosis	India	Treating drug-resistant tuberculosis in a low-intensity chronic conflict
McGuire, 2014 [27]	Review	Cardiovascular diseases	Low-resource settings	Medical devices and diagnostics for cardiovascular diseases
Weigl, 2014 [26]	Review	NCDs and chronic diseases	Low-resource settings	Point-of-care diagnostics and their impact on care in the age of the NCD and chronic disease epidemic
Doocy, 2015 [33]	Cross-sectional survey	Chronic diseases	Jordan	Prevalence and care-seeking for chronic diseases among Syrian refugees
Knaul, 2015 [24]	Review	Breast cancer	Mexico	Example of breast cancer care to illustrate effective universal health coverage along the chronic disease continuum and across health systems functions
Lee, 2015 [32]	Commentary	HIV	LMICs	Transition from paediatric to adolescent to adult healthcare settings for young HIV patients
Rabkin, 2016 [34]	Review	HIV/chronic diseases	LMICs	Lessons from HIV to address chronic diseases in protracted emergencies
Silverman-Retana, 2016 [29]	Cross-sectional survey	Diabetes mellitus/ hypertension	Mexico	Exploring transition of diabetes and hypertension care among male prisoners
Nobrega, 2017 [31]	Qualitative study	Chronic diseases	Brazil	Evaluating continuity of care for children and adolescents with chronic diseases in the healthcare network
Group 2: Mentioning continuity or lack of continuity without a detailed definition				
Greenberg, 2002 [53]	Commentary	Chronic diseases	LMICs	A new perspective on global health assistance given health transitions and rise of chronic illnesses
Polanczyk, 2009 [54]	Review	Coronary artery disease	Brazil	Contemporary management and future perspectives for coronary artery disease
Suwanno, 2009 [50]	Cross-sectional survey	Heart failure	Thailand	Predicting health status of a patient with heart failure
Ramli, 2010 [36]	Review	Chronic heart failure	Malaysia	Management of chronic heart failure in primary care
van Olmen, 2011 [55]	Commentary	Chronic diseases	LICs	Self-management facilitated by expert patient networks and smartphone technology
Lund, 2012 [51]	Review	Mental health	South Africa	Mental health services
Bhojani, 2013 [37]	Qualitative in-depth interviews	Diabetes mellitus	India	Patients perspective on managing diabetes care
Dasgupta, 2014 [52]	Commentary	Chronic malnutrition	India	Examining the burden of severe malnutrition (acute and chronic) and whether programmatic responses are consistent with epidemiologic realities
Ravaghi, 2014 [38]	Qualitative SSIs	Diabetes mellitus	Iran	Provider’s perspective on specialised care programme for diabetes
Atwine, 2015 [47]	Qualitative FGDs	Diabetes mellitus	Uganda	Health-seeking behaviour and use of traditional medicine among persons with type 2 diabetes
Hussein, 2015 [39]	Review	Diabetes mellitus	Malaysia	Status of diabetes care and management
Mahomed, 2015 [49]	Quasi-experimental study	Chronic diseases	South Africa	A multifaceted intervention to improve the quality of nurse clinical documentation for chronic patients at primary care clinics
Maimela, 2015 [40]	Qualitative study	Chronic diseases	South Africa	Perceptions and perspectives of patients and healthcare providers on chronic disease management
Malan, 2015a [41]	Qualitative interviews and FGDs	NCDs	South Africa	A situational analysis of training for behaviour change counselling for primary care providers
Malan, 2015b [42]	Qualitative study	NCDs	South Africa	Experiences of primary care providers after a training programme to offer brief behaviour change counselling on risk factors for NCDs
Puspitasari, 2015 [43]	Qualitative in-depth SSIs	NCDs	Indonesia	Challenges in the management of chronic NCDs by community pharmacists
Sellappans, 2015 [48]	Qualitative FGDs	Chronic diseases	Malaysia	Challenges faced by primary care physicians in a teaching hospital when prescribing for patients with chronic diseases
Wang, 2015 [44]	Household survey	Chronic NCDs	Malawi	Health-seeking behaviour and the related household out-of-pocket expenditure for chronic NCDs
Khodaveisi, 2017 [45]	Randomised clinical trial	Multiple sclerosis	Iran	Effect of continuous care on the lifestyle of patients with multiple sclerosis
Pelcastre-Villafuerte, 2017 [46]	Ethnographical review	Diseases among the elderly	Mexico	A comprehensive healthcare model, interculturally appropriate, designed to meet the needs of indigenous older adults
Group 3: Researching continuity of care in HIV/AIDS programmes and scaling them up to support NCD management				
Rabkin, 2011a [61]	Commentary	HIV/NCDs	LMICs	Leveraging HIV programmes to support NCD services
Rabkin, 2011b [62]	Commentary	HIV/NCDs	LMICs	Leveraging HIV programmes to support NCD services
Rabkin, 2012a [64]	Commentary	HIV/NCDs	LICs	Leveraging HIV programmes to support NCD services
Rabkin, 2012b [63]	Assessments and pilot intervention	HIV/diabetes mellitus	Ethiopia and Swaziland	Leveraging HIV programmes to support diabetes services
Fujita, 2015 [58]	Collaborative case study	HIV	6 Asia and Pacific countries	HIV service delivery model
Mkwinda, 2016 [56]	Qualitative design	HIV	Malawi	Exploring the needs of people living with HIV concerning care received from primary caregivers and palliative care nurses
Panditrao, 2015 [60]	Cross-sectional survey	HIV	India	Barriers to continued care among HIV-infected women who were previously enrolled in a private sector preventing mother-to-child transmission programme
Kruk, 2016 [59]	Discrete choice experiment	HIV	Ethiopia and Mozambique	Identifying healthcare characteristics preferred by HIV-infected women to promote treatment for a lifetime
Ahonkhai, 2017 [57]	Cross-sectional survey	HIV	Nigeria	Patient-centred medical home to provide HIV care
Group 4: Researching continuity of care in NCD management				
Arevian, 2005 [65]	Case study	Diabetes mellitus	Lebanon	Collaborative practice model delivering care for diabetes mellitus patients
Wei, 2008a [67]	Case study	Diabetes mellitus	China	Diabetes management programme and association of continuity of care with clinical outcomes
Hanafi, 2015 [68]	Retrospective cohort study	Hypertension	Malaysia	Impact of personal continuity of care on blood pressure control in a university-based primary care practice
Shi, 2015a [73]	Case-control study	Hypertension /diabetes	China	Impact of an integrated care delivery intervention on healthcare seeking and outcomes for chronically ill patients (i.e. with hypertension or diabetes)
Shi, 2015b [71]	Case-comparison study	Hypertension /diabetes	China	Examining which of the dominant primary care delivery models (i.e. public community health centres model, ‘gate-keeper’ CHC model or hospital-owned CHC model) was most effective in enhancing access to and quality of care for patients with chronic diseases (i.e. with hypertension or diabetes)
Tang, 2015 [66]	Study design	Hypertension	China	Study design of a clustered randomised controlled trial to build and evaluate an integrated healthcare system for chronic patients
Wei, 2015 [72]	Multistage stratified random survey	Chronic diseases	China	Changes in perspectives of patients on quality of primary care following the introduction of health system reforms
Mwangome, 2016 [75]	Qualitative in-depth interview	HIV/diabetes mellitus	Tanzania	Perceptions, experiences and practice of care for HIV and diabetes from the perspective of patients and family caregivers
Ye, 2016 [70]	Cohort study	Hypertension	China	Effect of continuity of care on health-related quality of life in adult patients with hypertension
Mwangome, 2017 [74]	Qualitative study	Diabetes mellitus	Tanzania	Perception of health providers on diabetes care provision
Zhang, 2017 [69]	Clustered randomised controlled trial	Hypertension	China	Effects of integrated chronic care models on hypertension outcomes and spending
Group 5: Measuring continuity of care with validated questionnaires				
Wei, 2008b [76]	Cross-sectional survey	Diabetes mellitus	China	Continuity of care in a community diabetes programme
Vargas, 2017 [77]	Cross-sectional survey	Chronic diseases	Columbia and Brazil	Patient perceptions of continuity of healthcare and associated factors

*CHC* community health center, *FGDs* focus group discussions, *LICs* low-income countries, *LMICs* low- and middle-income countries, *NCDs* non-communicable diseases, *SSIs* semi-structured interviews

^a^Location could be: a country or a region or a setting

The selected articles included 18 (32.7%) reviews or commentaries; 13 (23.6%) qualitative studies; 12 (21.8%) articles describing interventions to manage chronic diseases; 10 (18.2%) surveys; and 2 (3.6%) cohort studies. Half of the reviews or commentaries were region or country specific. Half of the qualitative studies were disease specific.

A geographical location was specified for 46 articles, as Asia (*n* = 25; 54.4%), Africa (*n* = 14, 30.4%) or South America (*n* = 7, 15.2%). The largest proportion of the articles from Asia referred to China (*n* = 8), followed by Malaysia (*n* = 4) and India (*n* = 4). All of the articles from China were quantitative studies. Most of the articles from Africa referred to South Africa (*n* = 5) and Tanzania (*n* = 3). The articles from South America were observational; there were no intervention studies. The main three categories of investigated conditions were either not specified (*n* = 19, 34.6%), diabetes mellitus or hypertension or both (*n* = 15, 27.3%), or HIV (*n* = 12, 21.8%), with half of these (*n* = 6) connecting HIV/AIDS care to other NCDs.

### List of used keywords across articles

Different keywords and word combinations were used to reflect the concept of CoC; four groups accommodated these keywords, as follows:

1. continued/continuous/continuity (i.e. continuity of patient care, continuity of healthcare, personal continuity of care, continuity of care record, care continuity, continued care, or continuous care);
2. continuum (i.e. continuum of care, continuum of care from prevention to treatment, continuum of disease care (e.g. continuum of HIV care), care continuum, chronic disease continuum or chronic disease care continuum);
3. continuity (i.e. coordination or integrated care); and
4. lack of continuity (i.e. discontinuity or fragmentation).

### Identified groups

The articles were divided into five groups based on their use of the CoC concept, namely (1) reflecting a change across stages or systems of care (*n* = 13; 24%), (2) mentioning continuity or lack of continuity without a detailed definition (*n* = 20; 36%), (3) researching CoC in HIV/AIDS programmes and its scaling up to support NCD management (*n* = 9; 16%), (4) researching CoC in NCD management (*n* = 11; 20%), and (5) measuring CoC with validated questionnaires (*n* = 2; 4%). The results about how the CoC was described are presented below, and analysed under each group of articles.

#### Group 1. Reflecting a change in disease stages or systems of care

Six reviews defined care for chronic illnesses as the provision of services across the continuum of care. Chronic care services contained primary prevention, secondary prevention, diagnosis, treatment, management, complication detection, survivorship, rehabilitation care, and palliative and end-of-life care [23–28]. This approach reflects services needed with changes in disease stages; most of the early services are available at the primary healthcare level, while advanced services are provided at secondary levels.

Two different settings used continuity as a concept in the context of ‘transition of care’, namely chronic disease care and management for prisoners to ensure consistent transition of care from community to prison and back [29] and the transition of care for children with chronic diseases moving from paediatric to adolescent to adult clinical settings [30–32]. The children’s transition process was described either as a CoC in two empirical studies from Brazil and Thailand [30, 31], or as a continuum of care in one review [32]. The qualitative research from Brazil evaluated CoC for children and adolescents from the perspectives of their families, healthcare providers and managers of local healthcare networks [31]. The authors defined CoC as “*an aspect of care experienced over time by individuals who use health care services and is determined by the integration ability of these services to coordinate health care actions coherently*” [31]. Central aspects discussed by participants included access to healthcare networks, community-based versus hospital-based care, continuity of information, referral systems, development of one unified treatment plan to be followed up by specialists and family doctors, and availability of resources, including medications.

Three articles mentioned CoC for chronic diseases in emergency settings. In emergencies, CoC delivery faces additional challenges with a mobile or traumatised population and a broken health system [33, 34]. Access was an essential precondition for treatment, follow-up and retention [33–35]. Rabkin et al. [34] discussed challenges in providing adequate care for NCDs in emergencies and ensuring continuity, which was defined as “*the need to deliver coordinated services over time*”. The types of interventions used by HIV/AIDS programmes that could support the provision of chronic care during emergencies were summarised. Examples included paper-based or electronic appointment systems, patient-held records, peer educators and patient support groups, and mobile phone apps and text reminders to facilitate retention.

#### Group 2. Mentioning continuity or lack of continuity without a detailed definition

This group discussed the concept of CoC as a critical component of chronic disease management, in addition to its importance for long-term outcomes; it only appeared in the introduction or discussion sections without a specific definition. The 20 articles researched different levels of the healthcare system, including system analysis (*n* = 7), provider perspectives (*n* = 6), patient perspectives (*n* = 6), and both provider and patient perspectives (*n* = 1).

Table 2 provides a summary of how the concepts of CoC were used. Continuity, defined as care delivered over time, was mentioned in more than half the articles (*n* = 11) and was less likely to appear in articles using system analysis [36–46]. Four reports defined lack of continuity as care provided by several health providers [40, 43, 47, 48], while four focused on continuity as the quality of the patient–professional relationship [40, 41, 43, 45]. All papers focusing on the consistency of providers or the patient–professional relationship were reporting patient or provider perspectives. Eleven articles focused on continuity as the coordination of care across levels and disciplines [36–39, 41, 45, 46, 49–52]; only two of the provider perspective articles discussed the informational component of continuity in particular [41, 49]. Finally, six articles discussed continuity with attention to adherence and compliance to treatment [47, 53, 54], access and availability of care [40, 46], or quality of care [55]. Detailed segments retrieved from articles for each CoC item are available in Additional file 3.

**Table 2:** Summary of the use of the ‘continuity of care’ concept among articles of Group 2 that mentioned continuity or lack of continuity without a detailed definition, divided by the articles’ nature of analysis (*n* = 20)

Items of continuity of care	Total (*n* = 20)	System analysis (*n* = 7)	Provider perspectives (*n* = 6)	Patient perspectives (*n* = 6)	Provider and patient perspectives (*n* = 1)
Longitudinal care (over time)	11 (55%)	2 (29%)	4 (67%)	4 (67%)	1 (100%)
Longitudinal care (consistency of personnel)	4 (20%)	/	1 (17%)	2 (33%)	1 (100%)
Patient–provider relationship	4 (20%)	/	1 (17%)	2 (33%)	1 (100%)
Coordinated care (across levels and disciplines)	11 (55%)	4 (57%)	4 (67%)	3 (50%)	/
Other (access, quality and adherence)	6 (30%)	3 (43%)	2 (33%)	/	1 (100%)

#### Group 3. Researching CoC in HIV/AIDS programmes and scaling them up to support NCD management

All identified articles defined CoC for HIV/AIDS patients as a follow-up to provide continuous life-long care [56–64]. Four empirical studies investigated CoC for HIV/AIDS patients from the patient perspective, and compared HIV service delivery models in different countries. These empirical studies mentioned other components of CoC, for example, healthcare access, linkages across levels and services, and quality of care [56–58, 60], in addition to recording systems and documentation transitions [57, 58].

The remaining four articles were reviews on contributions of HIV programmes as models for interventions addressing NCDs, given that both are chronic [61–64]. They defined CoC as “*coordination of services over time and across disciplines*” [64]; other dimensions of CoC were used but not explicitly mentioned in their definition, such as consistency of personnel [61] and patient–professional relationships [61, 62, 64]. The authors also stressed that approaches to integrate services for HIV and other diseases might vary: “*In some contexts, integration of services for all chronic diseases, HIV and NCD alike, may be the best approach. In others, programs may not be integrated at the point of service but may draw upon similar systems, from monitoring and evaluation to procurement*” [62]. The authors provided practical examples for successful HIV interventions with chronic care components relevant for other NCDs, such as counselling and adherence support, standardised treatment protocols, and task-shifting/task-sharing [61, 62].

#### Group 4. Researching CoC in NCD management

This group includes articles that mentioned CoC in their objectives or methods or as part of a NCD programme or intervention. In six articles, four interventions were described that improve CoC for patients with chronic diseases [65–70]. Three articles investigated one intervention in China, one discussing a proposal [66] and two on empirical datasets [69, 70]. Three other interventions were implemented at the primary healthcare level in China using two models, namely Starfield’s model of primary care and the Primary Care Assessment Tool; both have components of CoC [71–73]. Finally, two qualitative studies investigated CoC in the Tanzanian Health System at the patient and provider levels, based on WHO’s ICCC Framework [74, 75].

These 11 articles can be divided into two groups based on their objectives, namely those that aim to study CoC concerning access or quality (*n* = 6; 55%) [65, 66, 71, 72, 74, 75] and those aiming to investigate the impact of CoC on outcomes (*n* = 5; 45%) such as clinical outcomes [67, 68], healthcare seeking and clinical outcomes [73], healthcare spending and clinical outcomes, [69] or quality of life [70].

Table 3 provides a summary of how these articles used the concepts of CoC. All 11 articles included a component for longitudinal care over time with one healthcare setting or provider as a primary point of contact (e.g. community health centre, personal doctor or public health officer at a clinic/centre). Two interventions facilitated patients’ getting scheduled appointments.

**Table 3:** Summary of the use of the ‘continuity of care’ concept among articles of Group 4 that researched continuity of care in non-communicable disease management, divided by the articles’ type and use of conceptual models (*n *= 11, two articles of same study)

Characteristics	Total (*n* = 10)	Intervention (*n* = 5)	PHC model (*n* = 3)	ICCC framework (*n* = 2)
Data collection level				
System	4 (40%)	4 (80%)	0	0
Providers	1 (10%)	0	0	1 (50%)
Patients	5 (50%)	1 (20%)	3 (100%)	1 (50%)
Access	8 (80%)	3 (60%)	3 (100%)	2 (100%)
Quality	6 (60%)	1 (20%)	3 (100%)	2 (100%)
Measurements				
Quantitative measure for CoC^a^	8 (80%)	5 (100%)	3 (100%)	NA
Disease outcome	4 (40%)	4 (80%)	0	NA
Other outcomes^b^	3 (30%)	1 (20%)	2 (67%)	NA
Intervention components for providers				
Training for providers	5 (50%)	3 (60%)	2 (67%)	NA
Financial incentives	3 (30%)	2 (40%)	1 (33%)	NA
Items of continuity of care				
Longitudinal care (over time)	10 (100%)	5 (100%)	3 (100%)	2 (100%)
Longitudinal care (consistency of personnel)	6 (60%)	4 (80%)	2 (67%)	/
Patient–provider relationship	6 (60%)	2 (40%)	2 (67%)	2 (100%)
Coordinated care (across levels and disciplines)	7 (70%)	3 (60%)	3 (100%)	1 (50%)
Coordinated care (informational component)	6 (60%)	3 (60%)	2 (67%)	1 (50%)

*CoC* continuity of care, *ICCC* innovative care for chronic conditions, *NA* not available, *PHC* primary healthcare

^a^Examples were clinic utilisation, Likert scale, Usual Provider Continuity Index, Continuity of Care Index

^b^Examples were satisfaction, cost and ‘subjective’ health improvement, and quality of life

The second component of CoC usually included coordinated care across levels, for example, using team collaboration, standardised guidelines, referrals and clinical pathways, besides an informational component using medical records and information sharing, which appeared in the qualitative study. In most articles, there was no measurement or comprehensive explanation of aspects of coordinated care.

Articles that used primary healthcare models investigated the patient–provider relationship using proxy questions, for example, “*Healthcare professionals always encourage you to ask questions*” [71, 73]. Both patients and providers in the qualitative studies discussed the nature of their relationship, especially its role in patient education, improvement of illness management skills, and encouragement of self-management. Further information on definitions and measurements used in each article is given in Additional file 3.

#### Group 5. Measuring COC with validated questionnaires

Two articles used tools to measure the CoC from the patient’s perspective [76, 77]. One developed and validated a tool, while the other used an existing questionnaire. The instruments used two different definitions and frameworks for CoC.

Wei et al. [76] used Donaldson’s CoC definition based on agency theory [78], namely “*The degree to which health care activities are structured to increase information transfer and goal alignment between providers and patients to minimise agency loss*”. This assessment instrument for diabetic patients used two validated tools – the Primary Care Assessment Survey, after dropping ‘access’, and the Summary of Diabetes Self-care Activities Measure, comprising 46 items using a Likert-scale in the two sections of information transfer and goal alignment. The new tool was compared to a conventional measure of CoC, ‘Concentration of Care’ defined as “*the duration of care between the patient and his/her primary doctor and the proportion of total visits to the primary doctor*” [76].

Vargas et al. [77] determined the level of CoC perceived by users and explored influencing factors in two countries with different health systems (Colombia and Brazil). They used the CoC definition of Reid et al., namely “*the patient’s experience of care over time as connected and coherent with his or her health needs and personal circumstances. In other words, CoC refers to the perception and experience of an individual patient, as opposed to the providers’ perspective, which would be defined as coordination of care*” [79]. According to this definition, there are three types of CoC, namely (1) continuity of information, or the perception of transfer of clinical information across levels of care; (2) continuity of clinical management, or the perception of care coherence across levels of care; and (3) relational continuity, or the perception of an ongoing doctor–patient relationship.

The researchers used a validated tool, the CCAENAV questionnaire (*Cuestionario de Continuidad Asistencial Entre Niveles de Atencion,* in Spanish). The care continuity scale had four synthetic indexes for each of the following sub-scales: information transfer, care coherence, patient–primary care doctor relationship, and patient–secondary care doctor relationship, in addition to a separate item outside the scale for the consistency of health professionals. The questionnaire included 14 questions with a Likert-scale format.

There were five common themes in the questionnaires of Wei et al. [76] and Vargas et al. [77], namely (1) the consistency of health professionals; (2) primary care doctor’s knowledge of patient’s medical/clinical history; (3) primary care doctor provides counselling to patients using effective communication; (4) trust between provider and patient; and (5) coherence in treatment provided by primary care doctors and specialists reflecting collaboration across care levels.

The questionnaire of Wei et al. [76] included questions regarding the interpersonal relationship between provider and patients. For example, the primary care doctor’s knowledge of the patient’s responsibilities at home or work. A detailed comparison between the questionnaires of Wei et al. [76] and Vargas et al. [77] is available in Additional file 3.

## Discussion

The literature discussing CoC in LMICs has increased over the last two decades. Most articles suggested CoC as a priority for problems of people with chronic conditions but rarely specified a definition for CoC. In many cases, the words ‘continuum’ and ‘continuity’ were used interchangeably. Linguistically, the Oxford Dictionary defines continuity as “[t]*he unbroken and consistent existence or operation of something over time; a state of stability and the absence of disruption; a connection or line of development with no sharp breaks*” [80], and continuum as “[a] *continuous sequence in which adjacent elements are not perceptibly different from each other, but the extremes are quite distinct*” [81]. The definition of continuum corresponds with looking at care as a continuum provided from birth to end of life, or as services provided through the course of disease with a consecutive sequence of primary prevention, secondary prevention or early detection (e.g. screening), diagnosis, treatment, management and complication detection, survivorship and rehabilitation care, to palliative and end-of-life care. In this sense, although care as a continuum can help to evaluate the accessibility of the health system, it would be less critical for the patient’s perspective regarding their healthcare needs.

The concept of CoC was defined and used differently in various articles, complicating any synthesis of evidence related to CoC and its impact on long-term care. This observation is similar to the findings of a literature review that focused on research from high-income countries, which found that definitions of CoC and related concepts varied over time, leading to confusion [8, 82]. To avoid this ambiguity, we will use the definition of CoC as the provision of coordinated care and services over time and across levels and disciplines, which is coherent with the patient’s health needs and personal circumstances. This definition combines three key components of CoC – longitudinal care, the patient–provider relationship and coordinated care, including information management. This definition includes all the above-mentioned shared themes for long-term care. Additionally, it includes contextualisation of CoC based on the patient’s and provider’s characteristics and service organisation [10, 83].

At the primary healthcare level, CoC mostly means responding to the needs of patients with chronic or life-long conditions. The patient’s needs for CoC are similar in limited-resource and high-income settings. However, more research and policy need to focus on developing interventions that can be implemented by leveraging available resources within the health system. For example, many LMICs face an additional challenge of meeting the health needs of chronic patients in mobile populations (e.g. displaced, refugees) or emergency settings. Secondly, although CoC is mostly applied to NCDs, it also applies to chronic communicable diseases and chronic malnutrition. Therefore, it is better to describe CoC within the context of long-term care, providing an opportunity for health policy and systems to combine or integrate experiences and resources to tackle both communicable diseases and NCDs instead of competing for funds. Thirdly, studies defining CoC as care experienced by individuals need to consider the roles of patients, providers, systems and policy in implementing and achieving CoC. Fourthly, research on CoC occasionally assumes that health services are accessible to patients. To fully understand CoC and translate this understanding into policy, it is essential to look at CoC within the overall context of health services, especially regarding access and quality.

The definitions and components of CoC used in research in LMICs show similarities and differences. There is a shared understanding that CoC contains a component for longitudinal care as repeated and regular visits to health services over time, perhaps best provided at the lowest level, i.e. primary healthcare. Longitudinal care may also include a component for patients to interact with as few providers as possible, which may be harder to achieve when primary healthcare services are not easily accessible or do not satisfy minimum quality standards. Despite the importance of the patient–provider relationship, it was rarely included in the definitions of CoC used, and only mentioned during patient’s interviews with a primary focus on the provider’s role in encouraging self-management. The nature of the patient–provider relationship may include three characteristics, namely familiarity, so providers are able to consider the patient’s personal and social context; patient’s trust in their providers; and knowledge co-production, which leads to better health literacy and self-management practices.

The second most frequently mentioned component for CoC was coordinated care, which could take place within one level or across different levels in the health system; it could be achievable through referrals and back-referrals. In many resource-constrained settings, it is not easy to implement team-based collaboration, but coordinated care should provide patients with consistent, unified treatment plans and medications, especially across levels, based on guidelines or management protocols. Another essential characteristic of coordinated care is patient information management, whether written or electronic, with the aims of record-keeping over time to accumulate knowledge of prior visits and sharing or transferring information within and across levels.

**Fig. 1:**
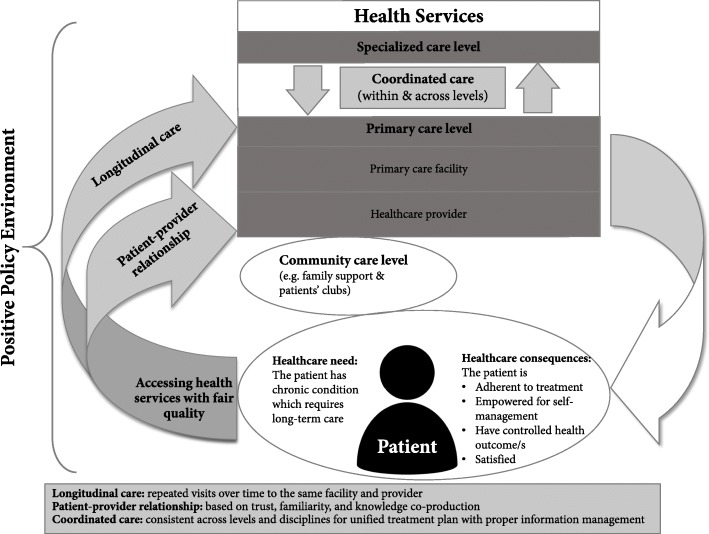
Conceptual framework for continuity of care and its three components as relevant to primary healthcare in a global context (including resource-constrained settings), adapted from WHO’s ICCC [7] and Salisbury 2009 [86]

Figure 1 illustrates the findings concerning CoC definitions and components, revealing the circular nature of long-term care. This framework can be useful in global health research, policy and planning. Even if the use of CoC focuses on one component, describing and understanding this framework ensures acknowledgement of other components and key contextual factors.

Interventions to promote longitudinal care may include paper-based or electronic appointment systems for scheduled follow-up, using appointment books, on-site medical records, or mobile phone applications. Nurturing a positive patient–provider relationship requires counselling during a consultation, and building partnerships with communities through peer educators and patient support groups. Promotion of mobile phone apps and text reminders may facilitate retention in care. Interventions to encourage information management include easy to carry or access patient-held records, possibly using mobile phone applications. To increase the consistency of care, standardised treatment protocols are crucial, customised to the context of health service delivery and health insurance. Other interventions to facilitate coordinated care may include task-shifting or task-sharing towards more available caregivers.

Finally, to achieve longitudinal care and a positive patient–provider relationship, most articles focused on strengthening primary healthcare, ensuring access, and the inclusion of chronic care in basic health benefit packages. These approaches build upon the Sustainable Development Goals, WHO’s global strategies towards people-centred and integrated health services [3], and universal health coverage [84]. The potential strategies target all building blocks of the health system, such as governance, financing, workforce, service delivery, information management, and medical products/technology. Current research and policy in resource-constrained settings aiming to identify and implement best practices to strengthen primary healthcare and extend universal health coverage should consider their role in achieving CoC, while providing a precise definition of the concept and its components.

The review has limitations. First, it involved one search engine (i.e. PubMed); however, it is unlikely that essential publications were missed given the review’s exploratory nature. Second, the processes of data abstraction and extraction were performed by a single researcher. However, the use of inductive thematic analysis matches a more robust strategy of a single researcher conducting data extraction at two separate time points [85].

In conclusion, those using the concept of CoC in research or policy must provide an explicit definition in their reports. A suggested definition of CoC is the provision of coordinated care and services over time and across levels and disciplines, which is coherent with patient’s health needs and personal circumstances. This definition covers three key components for CoC – longitudinal care (repeated visits over time), the nature of the patient–provider relationship (based on trust, familiarity and knowledge co-production), and coordinated care (consistent across levels and disciplines for a unified treatment plan with proper information management). This scoping review suggests a framework for CoC that could be operational for LMICs, acknowledging its multiple components and contextual factors, particularly access and quality. However, there is a need for further research and guidance to clarify and extend this definition and framework for emergency settings where CoC and its components are hard to achieve, especially repeated visits and positive patient–provider relationships, because of the limited patient mobility. The global priorities of strengthening primary healthcare and extending universal health coverage should consider interventions to achieve CoC, especially for patients with chronic conditions.

## Additional files

Additional file 1: Continuity of care concepts. Definitions for continuity of care with focus on primary healthcare settings (DOCX 87 kb)

Additional file 2: Search strategy. Detailed tables on the search terms and strategy used in PubMed (DOC 43 kb)

Additional file 3: Extra tables. Detailed segments retrieved for three of the five identified groups of articles. (DOC 187 kb)

## Abbreviations

CCM: Chronic Care Model
CoC: continuity of care
ICCC: Innovative Care for Chronic Conditions
LMICs: low- and middle-income countries
NCDs: non-communicable diseases
