# Supplementary material for: How have researchers defined and used the concept of ‘continuity of care’ for chronic conditions in the context of resource-constrained settings? A scoping review of existing literature and a proposed conceptual framework
Source: Health Res Policy Syst. 2019 Mar 7;17:27. doi: 10.1186/s12961-019-0426-1 (PMC6407241; doi:10.1186/s12961-019-0426-1)
Supplement: Supplementary file 1 — Continuity of care concepts. Definitions for continuity of care with focus on primary healthcare settings (DOCX 87 kb) [file 12961_2019_426_MOESM1_ESM.docx]

**Additional file 1:** Definitions for continuity of care with focus on primary healthcare settings

| **Oxford Dictionary** | |
| --- | --- |
| - *Continuity*: The unbroken and consistent existence or operation of something over time.   - A state of stability and the absence of disruption.   - A connection or line of development with no sharp breaks. - *Continuous*: Forming an unbroken whole; without interruption   - Forming a series with no exceptions or reversals. - *Continued*: Without a break in continuity; ongoing. - *Continuum*: A continuous sequence in which adjacent elements are not perceptibly different from each other, but the extremes are quite distinct. | |
| **Continuity of care in family practice** (Hennen, 1975) | |
| There are four dimensions of continuity of care in family practice: chronological, geographical, interdisciplinary, and interpersonal. | |
| **Continuity of care in family practice** (McWhinney, 1975) | |
| Continuity of care in family practice cannot be adequately described merely in terms of duration. It is not delineated by the nature of a patient's illness, but involves the family physician's ongoing commitment to the patient and his family as persons. An implicit contract exists between the family physician and the patient. This kind of continuity of care provides several important elements related to patient care, such as the feasibility of long-term observations allowing effective diagnosis with less need for extensive one-time workups, the potential for psychotherapy and counseling through a continuing personal relationship, and the opportunity to perceive illness in the context of the whole person and his environment. Continuity of responsibility by the family physician is achievable despite mobility of some patients and is more a matter of the physician's attitude and style of practice than duration of the doctor-patient relationship. | |
| **Chronic Care Model (CCM)** (Wagner, 1998) | |
| Patients and families struggling with chronic illness have different needs, and these needs are unlikely to be met by an acute care organization and culture. They require planned, regular interactions with their caregivers, with a focus on function and prevention of exacerbations and complications. This interaction includes systematic assessments, attention to treatment guidelines, and behaviorally sophisticated support for the patient’s role as self-manager. These interactions must be linked through time by clinically relevant information systems and continuing follow-up initiated by the medical practice. | |
| **Continuity in general practice** (Guthrie & Wyke, 2000) | |
| For general practitioners, continuity of care has traditionally meant that a patient visits the same doctor. What matters is personal continuity, in which an ongoing doctor-patient relationship ensures that care takes account of the patient’s personal and social context. By contrast, recent statements from the NHS Executive emphasise the importance of consistency and coordination of care.5 From this perspective, continuity can be enhanced by appropriate organisation, guidelines, and electronic medical records, irrespective of which doctor is seen. | |
| **Continuity of care in modern general practice** (G. K. Freeman, Olesen, & Hjortdahl, 2003; G. Freeman, Sheppard, Robinson, Ehrich, & Richards, 2001) | |
| The elements of continuity   1. *Experienced continuity*: the experience of a coordinated and smooth progression of care from the patient’s point of view;   To achieve this central element the service needs:   1. *Continuity of information*: excellent information transfer following the patient; 2. *Cross-boundary and team continuity*: effective communication between professionals and services and with patients; 3. *Flexible continuity*: to be flexible and adjust to the needs of the individual over time; 4. *Longitudinal continuity*: care from s few professionals as possible, consistent with other needs; 5. *Relational or personal continuity*: to provide one or more named individual professionals with whom the patient can establish and maintain a therapeutic relationship. | |
| **Innovations of Chronic Care Conditions (ICCC)** (WHO, 2002)  Micro-Level: Building Blocks at the Patient Interaction Level   - Prepared, informed, and motivated patients and families - Prepared, informed, and motivated health care teams - Prepared, informed, and motivated community partners   Meso-Level: Building Blocks for the Health Care Organization   - *Promote continuity and coordination* - Encourage quality care through leadership and incentives - Organize and equip health care teams - Support self-management and prevention - Use information systems   Meso-Level: Building Blocks for the Community   - Raise awareness and reduce stigma - Encourage better outcomes through leadership and support - Mobilize and coordinate resources - Provide complementary services   Macro-Level: Building Blocks for the Community   - Provide leadership and advocacy - Integrate policies - Promote consistent financing - Develop and allocate human resources - Support legislative frameworks - Strengthen partnerships | |
| - Promote continuity and coordination   - Patient with chronic conditions need services that are *coordinated* across levels of care (i.e. primary, secondary, and tertiary care) and across providers. Health care workers who care for the same patients need to communicate with each other. There is strength in the collective knowledge, information, and skills of multiple health care workers that far surpass that of a single provider.   Where possible, an identified “care coordinator” can serve as the overseer and director of a patient’s care ensuring that efforts of all involved health care workers are integrated and coordinated.   - - Care must be planned and thoughtful over the course of the condition. Follow-up visits should be scheduled and organizations must be proactive in caring for patients with chronic problems. Allowing symptoms or the onset of preventable complications to prompt patients to seek car is costly, inefficient, and ineffective. By contrast, planned care permits the early detection of complications and the swift identification of decline in patients’ health status. | |
| **Concepts and Measures of Continuity of Healthcare** (Reid, Haggerty, & McKendry, 2002) | |
| Concepts of Continuity | Measurements of Continuity |
| - *Continuity* can only exist as an aspect of care: • that is experienced by an individual; and • that is received over time. | Chronological measures   - Duration and intensity of patient/provider affiliation - Concentration of care among different providers   - Number of providers (same discipline) with whom a patient had contact during an episode of care or a specified time interval   - Usual provider of care (UPC index)   - Continuity of care (COC index) - Sequential care   - Sequential continuity (SECON index) |
| *Types of continuity* |  |
| - *Informational Continuity:* The availability and use of information on prior events and circumstances – be it other visits, laboratory results, referral recommendations, or informal care – to make current care appropriate for the individual and his or her condition (i.e. transfer of information; accumulated knowledge of patient) | - Transfer of information from one provider to another or from one organization or level of care to another - Uptake and use of information: whether providers are aware of what occurred previously and how this affects current care |
| - *Relational Continuity:* An ongoing therapeutic relationship/a sustained contact between a patient and one or more providers (is an undergirding that connects care over time); sustained contact between a patient and a provider (i.e. ongoing patient-provider relationship; consistency of personnel | - Affiliation: having a regular or personal physician or other providers - Strength of patient-provider inter-personal relationship (i.e. levels of communication, trust, comfort, overall knowledge about patient’s medical history, behavior, attitudes, preferences & social circumstances)   - Perception of continuity scale   - Components of primary care index   - Primary care assessment tool   - Primary care assessment survey: 1) interpersonal, patience, friendliness, caring, respect; 2) trust in provider; 3) extent to which patient feels known. |
| - *Management Continuity:* The provision of timely and complementary services within a shared management plan/The provision of separate types of healthcare over time in ways that complement each other so required services are not missed, duplicated, or poorly timed (i.e. consistency of care; flexibility) | - Prescribed follow-up   - Whether follow-up visits occur as scheduled   - Time to follow-up - Consistency of care across providers (~ overlaps with quality of care)   - How closely management protocols for specific diseases are followed when a patient’s treatment spans various settings and providers. |
| **A multidisciplinary review of continuity of care** (Haggerty et al., 2003) | |
| - *Continuity* is the degree to which a series of discrete healthcare events is experienced as coherent and connected and consistent with the patient’s medical needs and personal context - *Continuity of care* is distinguished from other attributes of care by two core elements—care over time and the focus on individual patients - Three types of continuity exist in all settings: informational, management, and relational   - *Informational continuity*—The use of information on past events and personal circumstances to make current care appropriate for each individual   - *Management continuity*—A consistent and coherent approach to the management of a health condition that is responsive to a patient’s changing needs   - *Relational continuity*—An ongoing therapeutic relationship between a patient and one or more providers - The emphasis on each type of continuity differs depending on the type and setting of care | |
| **Hierarchical Definition of Continuity of Care** (Saultz, 2003) | |
| - Informational: An organized collection of medical and social information about each patient is readily available to any health care professional caring for the patient. A systemic process also allows accessing and communicating about this information among those involved in the care - Longitudinal: In addition to informational continuity, each patient has a “medical home” where the patient receives most health care, which allows the care to occur in an accessible and familiar environment from an organized team of providers. This team assumes responsibility for coordinating the quality of care, including preventive services - Interpersonal: In addition to longitudinal continuity, an ongoing relationship exists between each patient and a personal physician. The patient knows the physician by name and has come to trust the physician on a personal basis. The patient uses this physician for basic health services and depends on the physician to assume personal responsibility for the patient’s overall health care. When the personal physician is not available, a coverage arrangement assures that longitudinal continuity occurs | |
| **What is ‘continuity of care’?** (Gulliford, Naithani, & Morgan, 2006) | |
| - *Continuous caring relationship*: with an identified health professional, and - *Seamless care*: which involves integration, coordination, and shared information between providers. | |
| **Quality Indicators of Continuity and Coordination of Care for Vulnerable Elder Persons** (Wenger & Young, 2007) | |
| 1. Identification of source of care 2. Medication follow-up in the outpatient clinic 3. Continuity of medication between physicians 4. Communication of reason for consultation 5. Communication of consultant recommendation at referent physician 6. Follow-up of diagnostic tests in the outpatient setting 7. Follow-up of medication after hospital discharge 8. Continuity of test results between venues of care 9. Medical visits and appointments after hospitalization 10. Follow-up after hospital hospitalization 11. Medical record transfer | |
| **The nature of informational continuity of care in general practice** (Agarwal & Crooks, 2008) | |
| The term continuity of care covers many different aspects of continuity, specifically informational, longitudinal, and relational/interpersonal aspects.5,6 Informational continuity is understood to be the availability of patient information to providers throughout a healthcare system. | |
| **How should continuity of care in primary health care be assessed?** (Salisbury, Sampson, Ridd, & Montgomery, 2009) | |
| Framework   - *Longitudinal continuity*: repeated consultations over time with as few doctors as possible; - *Patient-professional relationship*: caring relationship between the health professional and patient; - *Coordinated care*: seamless care between professionals and provider organizations – which encompasses: informational continuity, team continuity, and management continuity | *Measurements of Longitudinal Continuity*   - Concentration: the proportion of consultations with one specific provider; - Dispersion: the number of different professionals consulted; - Distribution: the distribution of consultations between providers, giving higher scores to people who consult fewer providers; - Sequence: whether each consultation was with the same provider as the previous consultation. |
| **Continuity of care and the patient experience: Quality of General Practice in England** (G. Freeman & Hughes, 2010) | |
| - *Relationship continuity*: a continuous therapeutic/caring relationship with a clinician; | - Identifying the usual doctor - Longitudinal continuity (e.g. UPC index, COC index) - Patient experience of relationship continuity (e.g. Patient questionnaires) |
| - *Management continuity*: continuity and consistency of clinical management, including providing and sharing information and care planning, and any necessary co-ordination of care required by the patient. | - Patient questionnaires - Audit of systems and working practices |
| **How unique is continuity of care?** (Uijen, Schers, Schellevis, & van den Bosch, 2012) | |
| - Continuity of care, coordination of care, integration of care, patient-centred care and case management all are concepts describing core qualities of care. Surprisingly, most concepts have changed their meanings and definitions substantially throughout the years and are conceptually entangled (Fig. 1). However, we found that researchers using one concept hardly ever refer to overlapping concepts. They seem to operate mainly within their own conceptual framework and literature. | |
| **Global strategy on people-centered and integrated health services** (WHO, 2015)  Five strategic directions:   1. Empowering and engaging people 2. Strengthening governance and accountability 3. Reorienting the model of care 4. Coordinating services 5. Creating an enabling environment | |
| *Care coordination:* a proactive approach in bringing care professionals and providers together around the needs of service users to ensure that people receive integrated and person-focused care across various settings;  *Collaborative care:* care that brings together professionals and/or organizations to work in partnership with people to achieve a common purpose;  *Continuity of care:* the degree to which a series of discrete health care events is experienced by people as coherent and interconnected over time and consistent with their health needs and preferences;  *Continuous care:* care that is provided to people over time across their life course;  *Co-production of health:* care that is delivered in an equal and reciprocal relationship between professionals, people using care services, their families and the communities to which they belong. Co-production implies a long-term relationship between people, providers and health systems when information, decision-making and service delivery become shared;  *Integrated health services:* the management and delivery of health services such that people receive a continuum of health promotion, disease prevention, diagnosis, treatment, disease-management, rehabilitation and palliative care services, through the different levels and sites of care within the health system, and according to their needs throughout the life course;  *People-centered health services*: an approach to care that consciously adopts the perspective of individuals, families, and communities, and sees them as participants as well as beneficiaries of trusted health systems that respond to their needs and preferences in humane and holistic ways. | |

Agarwal, G., & Crooks, V. A. (2008). The nature of informational continuity of care in general practice. *The British Journal of General Practice: The Journal of the Royal College of General Practitioners*, *58*(556), e17-24. https://doi.org/10.3399/bjgp08X342624

Freeman, G., & Hughes, J. (2010). *Continuity of care and the patient experience. An Inquiry into the Quality of General Practice in England*. The King’s Fund. Retrieved from https://www.kingsfund.org.uk/sites/default/files/field/field_document/continuity-care-patient-experience-gp-inquiry-research-paper-mar11.pdf

Freeman, G. K., Olesen, F., & Hjortdahl, P. (2003). Continuity of care: an essential element of modern general practice? *Family Practice*, *20*(6), 623–627.

Freeman, G., Sheppard, S., Robinson, I., Ehrich, K., & Richards, S. (2001). *Continuity of Care: Report of a Scoping Exercise for the NCCSDO*. London: National Co-ordinating Center for NHS Service Delivery and Organization. Retrieved from http://www.netscc.ac.uk/hsdr/files/project/SDO_ES_08-1009-002_V01.pdf

Gulliford, M., Naithani, S., & Morgan, M. (2006). What is “continuity of care”? *Journal of Health Services Research & Policy*, *11*(4), 248–250. https://doi.org/10.1258/135581906778476490

Guthrie, B., & Wyke, S. (2000). Does continuity in general practice really matter? *BMJ*, *321*(7263), 734. https://doi.org/10.1136/bmj.321.7263.734

Haggerty, J. L., Reid, R. J., Freeman, G. K., Starfield, B. H., Adair, C. E., & McKendry, R. (2003). Continuity of care: a multidisciplinary review. *BMJ : British Medical Journal*, *327*(7425), 1219–1221.

Hennen, B. K. (1975). Continuity of care in family practice. Part 1: dimensions of continuity. *The Journal of Family Practice*, *2*(5), 371–372.

McWhinney, I. R. (1975). Continuity of care in family practice. Part 2: implications of continuity. *The Journal of Family Practice*, *2*(5), 373–374.

Reid, R., Haggerty, J., & McKendry, R. (2002). *Defusing The Confusion: Concepts and Measures of Continuity of Healthcare*. Ottowa: Canadian Health Services Research Foundation. Retrieved from http://www.cfhi-fcass.ca/Migrated/PDF/ResearchReports/CommissionedResearch/cr_contcare_e.pdf

Salisbury, C., Sampson, F., Ridd, M., & Montgomery, A. A. (2009). How should continuity of care in primary health care be assessed? *The British Journal of General Practice: The Journal of the Royal College of General Practitioners*, *59*(561), e134-141. https://doi.org/10.3399/bjgp09X420257

Saultz, J. W. (2003). Defining and measuring interpersonal continuity of care. *Annals of Family Medicine*, *1*(3), 134–143.

Uijen, A. A., Schers, H. J., Schellevis, F. G., & van den Bosch, W. J. H. M. (2012). How unique is continuity of care? A review of continuity and related concepts. *Family Practice*, *29*(3), 264–271. https://doi.org/10.1093/fampra/cmr104

Wagner, E. H. (1998). Chronic disease management: what will it take to improve care for chronic illness? *Effective Clinical Practice: ECP*, *1*(1), 2–4.

Wenger, N. S., & Young, R. T. (2007). Quality indicators for continuity and coordination of care in vulnerable elders. *Journal of the American Geriatrics Society*, *55 Suppl 2*, S285-292. https://doi.org/10.1111/j.1532-5415.2007.01334.x

WHO. (2002). *Innovative care for chronic conditions: building blocks for action*. Geneva: World Health Organization.

WHO. (2015). *WHO global strategy on people-centred and integrated health services* (Interim Report). Geneva: World Health Organization, Service Delivery and Safety. Retrieved from http://www.who.int/servicedeliverysafety/areas/people-centred-care/global-strategy/en/
