# Supplementary material for: How have researchers defined and used the concept of ‘continuity of care’ for chronic conditions in the context of resource-constrained settings? A scoping review of existing literature and a proposed conceptual framework
Source: Health Res Policy Syst. 2019 Mar 7;17:27. doi: 10.1186/s12961-019-0426-1 (PMC6407241; doi:10.1186/s12961-019-0426-1)
Supplement: Supplementary file 2 — Search strategy. Detailed tables on the search terms and strategy used in PubMed (DOC 43 kb) [file 12961_2019_426_MOESM2_ESM.doc]

**Additional file 2:** Search Strategy

PubMed

| **No.** | **Query** |  |
| --- | --- | --- |
| #4 | #1 AND #2 AND #3 | 2,116 |
| #3 | "Developing Countries"[Mesh] OR (Deprived[tiab] AND (Countr*[tiab] OR Population*[tiab])) OR (Developing[tiab] AND (Countr*[tiab] OR Population*[tiab] OR Econom*[tiab] OR Nation*[tiab] OR World[tiab])) OR LAMI Countr*[tiab] OR (Less Developed[tiab] AND (Countr*[tiab] OR Population*[tiab] OR Econom*[tiab] OR Nation*[tiab] OR World[tiab])) OR (Lesser Developed[tiab] AND (Countr*[tiab] OR Nation*[tiab])) OR LMIC*[tiab] OR low middle income countr*[tiab] OR (Low Income[tiab] AND (Countr*[tiab] OR Population*[tiab] OR Econom*[tiab] OR Nation*[tiab] OR Setting*[tiab])) OR (Lower Income[tiab] AND (Countr*[tiab] OR Population*[tiab] OR Econom*[tiab] OR Nation*[tiab] OR Setting*[tiab])) OR (Middle Income[tiab] AND (Countr*[tiab] OR Population*[tiab] OR Econom*[tiab] OR Nation*[tiab] OR Setting*[tiab])) OR (Poor[tiab] AND (Countr*[tiab] OR Population*[tiab] OR Econom*[tiab] OR Nation*[tiab] OR World[tiab])) OR (Poorer[tiab] AND (Countr*[tiab] OR Population*[tiab] OR Econom*[tiab] OR Nation*[tiab] OR World[tiab])) OR Third World OR (Transitional[tiab] AND (Countr*[tiab] OR Econom*[tiab])) OR (under developed[tiab] AND (Countr*[tiab] OR Population*[tiab] OR Nation*[tiab] OR World[tiab])) OR (underdeveloped[tiab] AND (Countr*[tiab] OR Population*[tiab] OR Nation*[tiab] OR World[tiab])) OR (Under Served[tiab] AND (Countr*[tiab] OR Population*[tiab] OR Nation*[tiab] OR World[tiab])) OR (Underserved[tiab] AND (Countr*[tiab] OR Population*[tiab] OR Nation*[tiab] OR World[tiab])) OR resource limited setting*[tiab] OR limited resource setting*[tiab] OR resource constraint setting*[tiab] OR Afghanistan[tiab] OR Albania[tiab] OR Algeria[tiab] OR “American Samoa”[tiab] OR Angola[tiab] OR Armenia[tiab] OR Azerbaijan[tiab] OR Bangladesh[tiab] OR Belarus[tiab] OR Byelarus[tiab] OR Belorussia[tiab] OR Belize[tiab] OR Benin[tiab] OR Bhutan[tiab] OR Bolivia[tiab] OR Bosnia[tiab] OR Botswana[tiab] OR Brazil[tiab] OR Bulgaria[tiab] OR Burma[tiab] OR “Burkina Faso”[tiab] OR Burundi[tiab] OR “Cabo Verde”[tiab] OR “Cape verde”[tiab] OR Cambodia[tiab] OR Cameroon[tiab] OR “Central African Republic”[tiab] OR Chad[tiab] OR China[tiab] OR Colombia[tiab] OR Comoros[tiab] OR Comores[tiab] OR Comoro[tiab] OR Congo[tiab] OR “Costa Rica”[tiab] OR “Côte d'Ivoire”[tiab] OR Cuba[tiab] OR Djibouti[tiab] OR Dominica[tiab] OR “Dominican Republic”[tiab] OR Ecuador[tiab] OR Egypt[tiab] OR “El Salvador”[tiab] OR Eritrea[tiab] OR Ethiopia[tiab] OR Fiji[tiab] OR Gabon[tiab] OR Gambia[tiab] OR Gaza[tiab] OR “Georgia Republic”[tiab] OR Georgian[tiab] OR Ghana[tiab] OR Grenada[tiab] OR Grenadines[tiab] OR Guatemala[tiab] OR Guinea[tiab] OR “Guinea Bisau”[tiab] OR Guyana[tiab] OR Haiti[tiab] OR Herzegovina[tiab] OR Hercegovina[tiab] OR Honduras[tiab] OR India[tiab] OR Indonesia[tiab] OR Iran[tiab] OR Iraq[tiab] OR Jamaica[tiab] OR Jordan[tiab] OR Kazakhstan[tiab] OR Kenya[tiab] OR Kiribati[tiab] OR Korea[tiab] OR Kosovo[tiab] OR Kyrgyz[tiab] OR Kirghizia[tiab] OR Kirghiz[tiab] OR Kirgizstan[tiab] OR Kyrgyzstan[tiab] OR “Lao PDR”[tiab] OR Laos[tiab] OR Lebanon[tiab] OR Lesotho[tiab] OR Liberia[tiab] OR Libya[tiab] OR Macedonia[tiab] OR Madagascar[tiab] OR Malawi[tiab] OR Malay[tiab] OR Malaya[tiab] OR Malaysia[tiab] OR Maldives[tiab] OR Mali[tiab] OR “Marshall Islands”[tiab] OR Mauritania[tiab] OR Mauritius[tiab] OR Mexico[tiab] OR Micronesia[tiab] OR Moldova[tiab] OR Mongolia[tiab] OR Montenegro[tiab] OR Morocco[tiab] OR Mozambique[tiab] OR Myanmar[tiab] OR Namibia[tiab] OR Nepal[tiab] OR Nicaragua[tiab] OR Niger[tiab] OR Nigeria [tiab] OR Pakistan [tiab] OR Palau[tiab] OR Panama[tiab] OR “Papua New Guinea”[tiab] OR Paraguay[tiab] OR Peru [tiab] OR Philippines[tiab] OR Phillippines[tiab] OR Philipines[tiab] OR Phillipines[tiab] OR Principe[tiab] OR Romania[tiab] OR Rwanda[tiab] OR Ruanda[tiab] OR Samoa[tiab] OR “Sao Tome”[tiab] OR Senegal[tiab] OR Serbia[tiab] OR “Sierra Leone”[tiab] OR “Solomon Islands”[tiab] OR Somalia[tiab] OR “South Africa”[tiab] OR “South Sudan”[tiab] OR “Sri Lanka”[tiab] OR “St Lucia”[tiab] OR “St Vincent”[tiab] OR Sudan[tiab] OR Suriname[tiab] OR Swaziland[tiab] OR Syria[tiab] OR “Syrian Arab Republic”[tiab] OR Tajikistan[tiab] OR Tadzhikistan[tiab] OR Tadjikistan[tiab] OR Tadzhik[tiab] OR Tanzania[tiab] OR Thailand[tiab] OR Timor[tiab] OR Togo[tiab] OR Tonga[tiab] OR Tunisia[tiab] OR Turkey[tiab] OR Turkmen[tiab] OR Turkmenistan[tiab] OR Tuvalu[tiab] OR Uganda[tiab] OR Ukraine[tiab] OR Uzbek[tiab] OR Uzbekistan[tiab] OR Vanuatu[tiab] OR Vietnam[tiab] OR “West Bank”[tiab] OR Yemen[tiab] OR Zambia[tiab] OR Zimbabwe[tiab] | 1,089,296 |
| #2 | "Chronic Disease"[Mesh] OR "Comorbidity"[Mesh] OR chronic*[tiab] OR non communicable*[tiab] OR noncommunicable*[tiab] OR comorbidit*[tiab] OR co morbidit*[tiab] OR multimorbidit*[tiab] OR multi morbidit*[tiab] OR NCD*[tiab] OR longterm*[tiab] OR long term*[tiab] | 1,846,958 |
| [#1](https://www.ncbi.nlm.nih.gov/pubmed) | ((continuity[tiab] OR continuum[tiab] OR continu*[tiab]) AND care[tiab]) OR “continuum of care”[tiab] OR “continuity of care”[tiab] | 103,317 |
