# Supplementary material for: How have researchers defined and used the concept of ‘continuity of care’ for chronic conditions in the context of resource-constrained settings? A scoping review of existing literature and a proposed conceptual framework
Source: Health Res Policy Syst. 2019 Mar 7;17:27. doi: 10.1186/s12961-019-0426-1 (PMC6407241; doi:10.1186/s12961-019-0426-1)
Supplement: Supplementary file 3 — Extra tables. Detailed segments retrieved for three of the five identified groups of articles. (DOC 187 kb) [file 12961_2019_426_MOESM3_ESM.doc]

**Additional file 3**

**Table 1.** Detailed segments retrieved for each item of continuity of care from articles that mentioned continuity or lack of continuity without a detailed definition (n=20)

| **Level of analysis** | **Continuity of care aspects/items** |
| --- | --- |
|  | **Longitudinal (over time) 11** |
| System Analysis | - Continuity and quality of care is delivered for patients with chronic disease across time and settings (Ramli, Jackson, Toh, Ambigga, & Piterman, 2010) |
|  | - Health camps run by the clinics for the vicinity yield poor follow-ups and continuity of care (Hussein, Taher, Gilcharan Singh, & Chee Siew Swee, 2015) |
| Provider’s perspective | - Continuous follow-up (Ravaghi et al., 2014) |
|  | - Poor continuity of care; - Continuity of care, we never see the patient twice (Malan, Mash, & Everett-Murphy, 2015a) |
|  | - Poor continuity of care (Malan, Mash, & Everett-Murphy, 2015b) |
|  | - Long-term follow up with regular monitoring |
| Provider’s & patient’s | - The encounters between health care professionals and chronically ill patients may be variously sporadic or on-going, occasional or intensive, and may involve one primary care provider or an array of specialist practitioners. (Maimela et al., 2015) |
| Patient’s perspective | - Health camps or community outreach programmes, which were generally free of cost. However, such services were mainly ad-hoc diagnostic initiatives that were not linked to long-term, free healthcare services (Bhojani et al., 2013) |
|  | - … continuity of care options for people suffering from CNCDs (Wang et al., 2015) |
|  | - The continuous care model establishes and maintains a …. continuous communication (Khodaveisi et al., 2017) |
|  | - Access to continuous care (Pelcastre-Villafuerte et al., 2017) |
|  | **Longitudinal (consistency of personnel) 4** |
| Provider’s perspective | - Use of multiple pharmacies by patients also impedes the provision of continuity of care for chronic conditions (Puspitasari, Aslani, & Krass, 2015) |
|  | - These elderly patients may then be accompanied by different carers to different doctors for their follow-up visits at different healthcare institutions (Sellappans, Lai, & Ng, 2015) |
| Provider’s & patient’s | - The encounters between health care professionals and chronically ill patients may be variously sporadic or on-going, occasional or intensive, and may involve one primary care provider or an array of specialist practitioners. (Maimela et al., 2015) |
| Patient’s perspective | - Switching between health care providers (Atwine, Hultsjö, Albin, & Hjelm, 2015) |
|  | **Patient-professional relationship 4** |
| Provider’s perspective | - Both doctors and nurses expressed difficulties in counselling a patient when they themselves were smokers or overweight. Doctors felt that nurses had a better understanding of the patient’s language and culture because they often stay in the community (Malan et al., 2015a) |
|  | - Primary health care is the most appropriate setting for chronic care because of its relative proximity to the patients’ home enabling providers to easily deliver follow-up (Puspitasari et al., 2015) |
| Provider’s & patient’s | - Encounters between patients and their health care professionals become a critical intersection for information exchange, decision-making and motivation. The ability of the health care professional to engage in effective communication (Maimela et al., 2015) |
| Patient’s perspective | - Communication between the nurse and the patient (Khodaveisi et al., 2017) |
|  | **Coordinated 11** |
| System Analysis | - Primary care physicians must ultimately become the agent of change and lead multidisciplinary primary care teams (Ramli et al., 2010) |
|  | - The tertiary level, greater continuity of care with primary and secondary levels is required to prevent “revolving door” patterns of care (Lund, Petersen, Kleintjes, & Bhana, 2012) |
|  | - Continuum of care (CoC) is weak and fails to sustain weight gains made during the stay at the NRC. CoC requires considerable strengthening to make both hospital- and community-based interventions meaningful (Dasgupta, Sinha, & Yumnam, 2014) |
|  | - Multidisciplinary diabetes team could be formed - Some clinics in Malaysia have medical social workers as part of the diabetes team enabling continuity of care for this chronic disease (Hussein et al., 2015) |
| Provider’s perspective | - Maintain the health care system continuum by providing a referral feedback system from levels of specialized treatment back to PHC (Ravaghi et al., 2014) |
|  | - Information management (Mahomed, Naidoo, Asmall, & Taylor, 2015) |
|  | - Poor record keeping; - Lack of a standardised approach; - Nurses lacked the knowledge on risk factors and NCDs when counselling. This also led to the problem of HCPs giving conflicting or contradictory messages (Malan et al., 2015a) |
| Patient’s perspective | - Continuity care programs promoting self-management ability should be developed and implemented both in hospital-based and home-based settings (Suwanno, Petpichetchian, Riegel, & Issaramalai, 2009) |
|  | - Health camps or community outreach programmes, which were generally free of cost. However, such services were mainly ad-hoc diagnostic initiatives that were not linked to long-term, free healthcare services; - Patients had to visit multiple locations to receive care for a single medical encounter; - In hospitals, lack of coordination between doctors resulting in different opinions (Bhojani et al., 2013) |
|  | - The continuous care model establishes and maintains a dynamic, flexible. communication (Khodaveisi et al., 2017) |
|  | - Inter-institutional communication/interaction. Continuous feedback and follow-up mechanisms greatly strengthen this type of strategies involving community participation (Pelcastre-Villafuerte et al., 2017) |
|  | **Other 6** |
| System Analysis | - Assuring CoC is a component of chronic disease management, and that CoC and compliance are essential to long term outcomes (Greenberg & Farmer, 2002) |
|  | - Continuity of care is a problem for chronic patients, and have an effect on adherence to therapies and prevention programmes (Polanczyk & Ribeiro, 2009) |
|  | - Lack of quality and continuity of health care result in high health care expenditure and very poor health outcomes (van Olmen et al., 2011) |
| Provider’s & patient’s | - Lack of continuous availability of medicines plays an essential part in the provision of health care for chronic conditions (Maimela et al., 2015) |
| Patient’s perspective | - Sometimes taking herbal medicine you don’t continue with it after feeling better; - Discontinuity in taking herbal as well as western medicine was noted in some cases when people felt better (Atwine et al., 2015) |
|  | - Access to continuous care (Pelcastre-Villafuerte et al., 2017) |

**References.**

Atwine, F., Hultsjö, S., Albin, B., & Hjelm, K. (2015). Health-care seeking behaviour and the use of traditional medicine among persons with type 2 diabetes in south-western Uganda: a study of focus group interviews. *The Pan African Medical Journal*, *20*, 76.

Bhojani, U., Mishra, A., Amruthavalli, S., Devadasan, N., Kolsteren, P., De Henauw, S., & Criel, B. (2013). Constraints faced by urban poor in managing diabetes care: patients’ perspectives from South India. *Global Health Action*, *6*, 22258.

Dasgupta, R., Sinha, D., & Yumnam, V. (2014). Programmatic response to malnutrition in India, room for more than one elephant. *Indian Pediatrics*, *51*(11), 863–868.

Donaldson, M. S. (2001). Continuity of care: a reconceptualization. *Medical Care Research and Review: MCRR*, *58*(3), 255–290. https://doi.org/10.1177/107755870105800301

Greenberg, H. M., & Farmer, R. G. (2002). Global health assistance: a new perspective. *Annals of Noninvasive Electrocardiology : The Official Journal of the International Society for Holter and Noninvasive Electrocardiology, Inc*, *7*(1), 73–77.

Hussein, Z., Taher, S. W., Gilcharan Singh, H. K., & Chee Siew Swee, W. (2015). Diabetes Care in Malaysia: Problems, New Models, and Solutions. *Annals of Global Health*, *81*(6), 851–862.

Khodaveisi, M., Ashtarani, F., Beikmoradi, A., Mohammadi, N., Mahjub, H., Mazdeh, M., & Ashtarani, E. (2017). The Effect of Continuous Care on the Lifestyle of Patients with Multiple Sclerosis: A Randomized Clinical Trial. *Iranian Journal of Nursing and Midwifery Research*, *22*(3), 225–231.

Lund, C., Petersen, I., Kleintjes, S., & Bhana, A. (2012). Mental health services in South Africa: taking stock. *African Journal of Psychiatry*, *15*(6), 402–405.

Mahomed, O. H., Naidoo, S., Asmall, S., & Taylor, M. (2015). Improving the quality of nurse clinical documentation for chronic patients at primary care clinics: A multifaceted intervention. *Curationis*, *38*(1). Retrieved from https://www.ncbi.nlm.nih.gov/pubmed/26841914

Maimela, E., Van Geertruyden, J.-P., Alberts, M., Modjadji, S. E. P., Meulemans, H., Fraeyman, J., & Bastiaens, H. (2015). The perceptions and perspectives of patients and health care providers on chronic diseases management in rural South Africa: a qualitative study. *BMC Health Services Research*, *15*, 143.

Malan, Z., Mash, B., & Everett-Murphy, K. (2015a). A situational analysis of training for behaviour change counselling for primary care providers, South Africa. *African Journal of Primary Health Care & Family Medicine*, *7*(1). Retrieved from https://www.ncbi.nlm.nih.gov/pubmed/26245589

Malan, Z., Mash, R., & Everett-Murphy, K. (2015b). Qualitative evaluation of primary care providers experiences of a training programme to offer brief behaviour change counselling on risk factors for non-communicable diseases in South Africa. *BMC Family Practice*, *16*, 101.

Pelcastre-Villafuerte, B. E., Meneses-Navarro, S., Ruelas-González, M. G., Reyes-Morales, H., Amaya-Castellanos, A., & Taboada, A. (2017). Aging in rural, indigenous communities: an intercultural and participatory healthcare approach in Mexico. *Ethnicity & Health*, *22*(6), 610–630.

Polanczyk, C. A., & Ribeiro, J. P. (2009). Coronary artery disease in Brazil: contemporary management and future perspectives. *Heart (British Cardiac Society)*, *95*(11), 870–876.

Puspitasari, H. P., Aslani, P., & Krass, I. (2015). Challenges in the management of chronic noncommunicable diseases by Indonesian community pharmacists. *Pharmacy Practice*, *13*(3), 578.

Ramli, A., Jackson, B., Toh, C., Ambigga, D., & Piterman, L. (2010). Management of Chronic Heart Failure in Primary Care: What Evidence do we have for Heart Failure with Preserved Systolic Function? *Malaysian Family Physician : The Official Journal of the Academy of Family Physicians of Malaysia*, *5*(2), 68–76.

Ravaghi, H., Sajadi, H. S., Ghotbi, M., Sarvarizadeh, S., Sharbafchizadeh, N., & Kermanchi, J. (2014). Evaluation of an urban phase of the specialized care program for diabetes in iran: providers’ perspectives. *International Journal of Preventive Medicine*, *5*(8), 1013–1022.

Reid, R., Haggerty, J., & McKendry, R. (2002). *Defusing The Confusion: Concepts and Measures of Continuity of Healthcare*. Ottowa: Canadian Health Services Research Foundation. Retrieved from http://www.cfhi-fcass.ca/Migrated/PDF/ResearchReports/CommissionedResearch/cr_contcare_e.pdf

Sellappans, R., Lai, P. S. M., & Ng, C. J. (2015). Challenges faced by primary care physicians when prescribing for patients with chronic diseases in a teaching hospital in Malaysia: a qualitative study. *BMJ Open*, *5*(8), e007817.

Suwanno, J., Petpichetchian, W., Riegel, B., & Issaramalai, S.-A. (2009). A model predicting health status of patients with heart failure. *The Journal of Cardiovascular Nursing*, *24*(2), 118–126.

van Olmen, J., Ku, G. M., Bermejo, R., Kegels, G., Hermann, K., & Van Damme, W. (2011). The growing caseload of chronic life-long conditions calls for a move towards full self-management in low-income countries. *Globalization and Health*, *7*, 38.

Wang, Q., Brenner, S., Leppert, G., Banda, T. H., Kalmus, O., & De Allegri, M. (2015). Health seeking behaviour and the related household out-of-pocket expenditure for chronic non-communicable diseases in rural Malawi. *Health Policy and Planning*, *30*(2), 242–252.

**Table 2.** Definitions and measurements of continuity of care used in articles that researched continuity of care in non-communicable disease management (n=11)

| **First author, year** | **Definition/measurements of continuity of care (CoC)** |
| --- | --- |
| **CoC in an intervention** | |
| Averian, 2005 | CoC measured by the number of patients who were lost to follow up and an average number of clinic visits per year. |
| Wei, 2008 | CoC definition based on agency theory: the degree to which health care activities are structured to increase information transfer and goal alignment between patients and doctors. The term CoC reflected the extent to which doctors partnered with patients and hypothesized that it would be associated with health outcome improvements. The adapted and validated tool is discussed in Theme 5. |
| Hanafi, 2015 | Operationalized "continuity of care" as patients seeing the same personal doctor at each visit, measured using the usual provider continuity index (UPCI); while informational continuity was maintained through the means of paper-based medical records. |
| Tang, 2015  Ye, 2016  Zhang, 2017 | Focused on longitudinal continuity as defined by Saultz, which is a familiar setting for patients to receive care that is easier for patients to access. In other words, it focused on a long-term relationship for patients and physicians, without involving the sense of trust and responsibility. The measurement used was continuity of care index (COCI). |
| **CoC as a function of primary healthcare model** | |
| Shi, 2015  Shi, 2015 | Used Starfield’s model of primary care, which included four components of quality (i.e. accessibility, continuity, coordination, and comprehensiveness) and three components of value (i.e. satisfaction, cost and health improvement).  Items on continuity as a component of quality of care were: healthcare professionals 1) reviewed with you all the medications; 2) always encourage you to ask questions; 3) contacts you to see how things are going. |
| Wei, 2015 | Used Primary Care Assessment Tool, which measured 8 attributes: two for first-contact (utilization and accessibility), continuity of care, two for coordination (services and information), two for comprehensiveness (available service and provided service), and a measure of how well the care was patient-focused/ cantered.  For each attribute, patients were asked how well they agree with the statement that their regular provider of primary care is excellent in terms of the attribute. |
| **CoC as part of Innovative Care for Chronic Conditions (ICCC) Framework** | |
| Mwangome, 2016 | The role of patients and caregivers in promoting continuity of care according to ICCC framework. |
| Mwangome, 2017 | The role of healthcare organization for promoting continuity and coordination according to ICCC framework:   - Availability of services at the facility level - Services coordinated across levels of care from primary to tertiary - Services coordinated across providers—i.e. providers should communicate - Care coordinator to serve as overseer and director of patient care - Care planned over the course of a condition - Schedule follow-up visits for patients - Providers to be proactive in-patient care |

**Table 3.** Comparison between the questionnaires used to measure continuity of care

| **Wei, 2008** | **Vargas, 2017** |
| --- | --- |
| **Definition of continuity of care** | |
| CoC definition of Donaldson (Donaldson, 2001): “The degree to which health care activities are structured to increase information transfer and goal alignment between providers and patients in order to minimize agency loss”. | CoC definition of Reid (Reid, Haggerty, & McKendry, 2002): “the patient's experience of care over time as connected and coherent with his or her health needs and personal circumstances. In other words, continuity of care refers to the perception and experience of an individual patient, as opposed to the providers’ perspective, which would be defined as coordination of care”.  There were 3 types of continuity of care: 1) Continuity of information: perception of transfer of clinical information across levels of care; 2) Continuity of clinical management: perception of care coherence across levels of care; and 3) Relational continuity: perception of an ongoing doctor-patient relationship.  Continuity of clinical management and information can be analysed within and across levels of care (referring to the interaction between providers) whereas relational continuity can only be analysed in each care level separately |
| **Questionnaire’s structure** | |
| Two sections:  22 items: Information transfer  24 items: Goal alignment  All items (46 items) used a 6-point Likert-scale format, except trust which used a 5-point Likert-scale format | Care continuity scale had four synthetic indexes for each of the following sub-scales:  3 items: Information transfer (informational continuity)  3 items: Care coherence (management continuity)  4 items: Patient-primary care doctor relationship (relational continuity)  4 items: Patient-secondary care doctor relationship (relational continuity)  2 items: Outside the scale: consistency of health professionals (relational continuity)  All items (14 items) used a 4-point Likert-scale format (never/rarely/very often/always) |
| **Consistency of health professionals** | |
| Assumed that patients are using a primary care doctor. The conventional measure, the concentration of care used the duration of care between the patient and his/her primary doctor and the proportion of total visits to the primary doctor. | A question on the consistency of health professional (i.e. being seen by the same doctor), a dimension of relational continuity, was included, but not as an item in the scale because it did not group with any of the factors.   - Attended by the same primary care doctor - Attended by the same specialist for the same health problem |
| **Primary care doctor’s knowledge of patient’s medical/clinical history** | |
| As part of information transfer:  Contextual knowledge of the patient (from PCAS): Primary physician’s knowledge of patient’s medical history | As part of information transfer’s items: Knowledge of clinical history   - My primary care doctor is aware of the instructions given to me by the specialist before I explain them to him/her - The specialist is aware of the instructions given to me by my primary care doctor before I explain them to him/her |
| **Primary care doctor provides counselling to patients using effective communication** | |
| As part of information transfer’s items:  Personal communication (from PCAS): Thoroughness of primary physician’s patience and attention to patient’s explanation; clarity of physician’s explanations of diseases, instructions and their help in implementing them  Diabetes counselling (from SDSAM): Primary physician’s discussion of diet, physical exercises, stress control and their applicability in patient’s daily life | As part of information transfer’s items:  Delivery of opportune and adequate information to patients   - After seeing the specialist, my primary care doctor discusses the visit with me   and  Effective communication ﻿   - The information my primary care/secondary care doctor gives me is sufficient |
| **Trust between provider and patient** | |
| As part of goal alignment’s items:  Trust (from PCAS): Patient’s assessment of primary physician’s integrity, competence, and role as patient’s agent. | As part of patient-primary care doctor relationship and patient-secondary care doctor relationship's items: Trust between provider and patient,   - I have confidence in the professional ability of my primary care/secondary care doctor - I feel comfortable consulting my primary care/secondary care doctor about my concerns and health problems - I would recommend my primary care/secondary care doctor to my family and friends |
| **Coherence in treatment provided by primary care doctor and specialist** | |
| As part of goal alignment’s items:  Integration (from PCAS): Primary physician’s knowledge of patient’s treatment by other family doctors or specialists, | As part of care coherence’s items: Coherence of care across care levels   - My primary care doctor is in agreement with the specialist’s instructions - The specialists are in agreement with my primary care doctor’ instructions - Collaboration across care levels - I think that my primary care doctor collaborates with the specialist to solve my health problems |
| **Interpersonal relationship between provider & patient** | **Reasons for patient’s perception of continuity of care** |
| As part of information transfer’s items:  Contextual knowledge of the patient (from PCAS): Primary physician’s knowledge of patient's responsibility at home or work, main health concern, values, and beliefs,  As part of goal alignment’s items:  Interpersonal treatment (from PCAS): Primary physician’s patience, friendliness, caring respect and appropriateness of time spent with patients. | Open-ended questions were included on the reasons for their perceptions of continuity of care; for example, users’ reasons for perceiving that their primary care doctor collaborated with the specialists to solve their health problems (one of the care coherence items) were elicited by following up the item with the open-ended question ‘Why?’. They were analysed qualitatively using content analysis. |
| **Quality of care** |  |
| As part of information transfer’s items:  Personal communication (from PCAS): Thoroughness of primary physician’s questions on symptoms  As part of goal alignment’s items:  Physical examination (from PCAS): Thoroughness of primary physician’s physical examination,  Process of care (new): How often the primary doctor checked feet and made retinopathy referral. |  |

PCAS, Primary Care Assessment Survey; SDSAM, Summary of Diabetes Self-care Activities Measure
